# Natural conception in HIV-serodiscordant couples with the infected partner in suppressive antiretroviral therapy: A prospective cohort study

**DOI:** 10.1097/MD.0000000000004398

**Published:** 2016-07-29

**Authors:** Jorge Del Romero, María Begoña Baza, Isabel Río, Adrián Jerónimo, Mar Vera, Victoria Hernando, Carmen Rodríguez, Jesús Castilla

**Affiliations:** a Centro Sanitario Sandoval, Instituto de Investigación Sanitaria San Carlos -IdISSC; b Centro Nacional de Epidemiología, Instituto de Salud Carlos III, Madrid; c CIBER Epidemiología y Salud Pública; d Instituto de Salud Pública de Navarra, IdiSNA—Navarra Institute for Health Research, Pamplona, Spain.

**Keywords:** antiretroviral therapy, HIV infection, HIV transmission, natural conception, serodiscordant couples

## Abstract

The potential of antiretroviral treatment (ART) to prevent the sexual transmission of HIV has increased the number of serodiscordant couples who are considering natural conception. We aim to describe the results of a protocol for reproductive counseling aimed at HIV serodiscordant couples who desire natural conception, in which the infected partner, the index case, is receiving suppressive antiretroviral treatment.

A prospective cohort included all HIV serodiscordant couples attended a counseling program in the period 2002 to 2013 who opted for natural conception and met the following criteria: index case on ART with persistent plasma viral suppression for at least the previous 6 months, ART compliance over 95%, preserved immune status, undetectable HIV viral and proviral load in semen in male index cases, and absence of genitourinary infections and fertility problems in both members of the couple.

Of the 161 HIV serodiscordant couples included, 133 with male index cases, 66% achieved at least 1 pregnancy, 18% a second one, and 5% a third pregnancy. A total of 144 natural pregnancies occurred and 107 babies were born. The pregnancy rate was 1.9 for each 100 acts of vaginal intercourse, and the mean time to conception was 6.1 months, both independently of the sex of the index case. No case of sexual or vertical HIV transmission occurred.

In the absence of fertility problems and under controlled conditions, natural conception might be a safe and effective reproductive method for those HIV serodiscordant couples who choose this reproductive option.

## 1 Introduction

Antiretroviral treatment (ART) has achieved a drastic reduction in the morbidity and mortality of infected persons^[1]^ and in the risk of sexual and vertical HIV transmission.^[2–7]^ This has led to an increasing number of HIV-serodiscordant couples (SDCs) who wish to have children. Some investigators consider that unprotected sexual relations under controlled conditions may become a safe reproductive method,^[8]^ others argue that it is not possible to completely rule out a residual risk of sexual transmission of HIV,^[9]^ which would make assisted reproduction a safer option for these couples.^[10]^ Prophylaxis with antiretroviral drugs pre- and post-HIV exposure could, according to some authors, boost prevention in natural conception.^[11,12]^ The lack of studies supporting the preventive potential of ART in the context of natural reproduction hampers professional counseling about the most appropriate reproductive choices for HIV SDCs.^[13,14]^

The objective of our study was to describe the results of a protocol for reproductive counseling aimed at HIV SDCs who desire natural conception, in which the infected partner (index case) is receiving suppressive ART to provide new evidence to help decision making in patients and health professionals in this area.

## 2 Methods

In April 1989, a free public clinic for sexually transmitted diseases in Madrid launched a preventive intervention program aimed specifically at heterosexual HIV SDCs.^[15]^ As of December 2013, 930 couples had been treated and followed up in this program.

To respond to the growing demand for reproductive counseling in HIV SDCs, a clinical protocol was established in March 2002 to provide guidance on the most appropriate reproductive methods for each couple: natural reproduction, autoinsemination or assisted reproduction. The protocol for reproductive counseling included only SDCs who wished to have children, in whom the index case was receiving ART with treatment adherence over 95%. It consisted of the following analytical determinations: blood HIV1 RNA viral load (Versant kPCR from Siemens) and T CD4+ lymphocytes count (EPICS XL-MCL from Coulter) in index cases; semen HIV1 RNA viral load and HIV1 DNA proviral load (in-house designed procedures with Qiagen reagents for both isolations and PCR and one-step RT-PCR amplification of gag HIV1 conserved sequences) in male index cases; HIV1/2 serology (CMIA from Abbott) in contacts; uretral and cervical/vaginal exudates (microscopy/cultures/RealTime PCR m2000rt from Abbott), urine analysis (URI-Clip Test from Menarini), syphilis serology (Cromatest RPR from Linear/EIA from Trinity Biothech/Serodia TP-PA from Fujirebio Inc) and hepatitis A, B, and C viruses serologies (CMIA from Abbott), hematology (Sysmex XT 1800i) and biochemistry (Cobas c 311 Roche) in both members of the couple; spermiogram^[16]^ in males and urine luteinizing hormone qualitative test (Clearblue CEFM from Unipath Ltd) in females (Table 1).

**Table 1:**
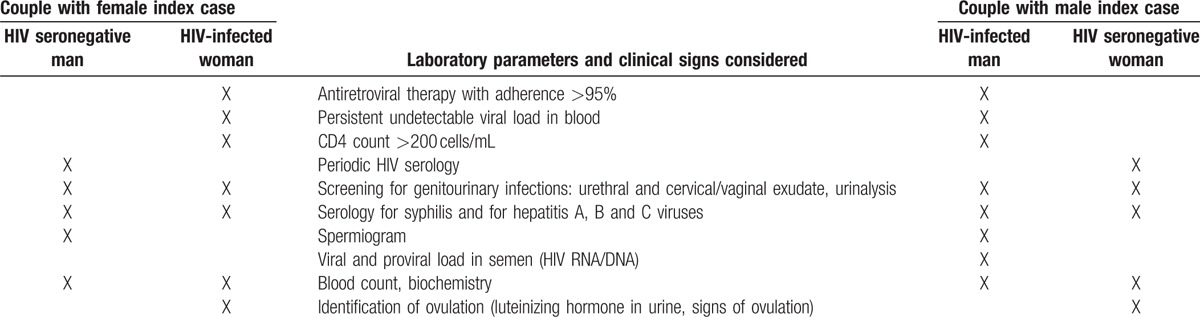
Laboratory parameters and clinical signs considered in each member of the HIV serodiscorant couple according to sex and HIV serological status.

The protocol of this study was approved by the Clinical Research Ethics Committee of the Hospital Clínico San Carlos in Madrid, Spain. The patients were informed about the aim of the study and they agreed to participate by signing an informed consent.

Alterations in semen parameters were defined according to the 1999 reference values of the World Health Organization.^[16]^

Based on the results of the protocol, the physician explained to each couple the reproductive options, so that they could decide the reproductive method to use. Those who opted for natural conception were reminded of the importance of absolute adherence to ART to have a sustained undetectable plasma viral load for at least the previous 6 months and were advised to limit their unprotected sexual relations to the periovulatory period; once pregnancy was achieved, they were told to resume systematic use of condoms. The serological status of the contact was reevaluated every 3 months or immediately when pregnancy was suspected and the number of unprotected vaginal intercourses was recorded.

Newborns of women with HIV infection were monitored to detect possible perinatal transmission in accordance with World Health Organization criteria.^[17]^

A descriptive analysis was made of the sociodemographic, behavioral, and clinical characteristics of each member of the couples that attempted natural reproduction between March 2002 and December 2013. We analyzed the epidemiological characteristics, clinical profile of the index case, total number of unprotected acts of vaginal intercourse and pregnancies achieved, as well as the final outcome of each (birth, voluntary interruption of pregnancy, or spontaneous abortion); these were compared by sex of the index case using the *χ*^2^ or Fisher exact test. The rates of pregnancy and of sexual HIV transmission were calculated per 100 and 1000 unprotected acts of vaginal intercourse, respectively, and the number of vertical transmissions was recorded. *P* value <0.05 was taken to indicate significance.

## 3 Results

Between 2002 and 2013, 198 HIV SDCs sought reproductive counseling. After being informed in accordance with the clinical protocol, 161 couples chose natural reproduction. The characteristics of the 37 couples who did not choose natural reproduction are shown in Table 2.

**Table 2:**
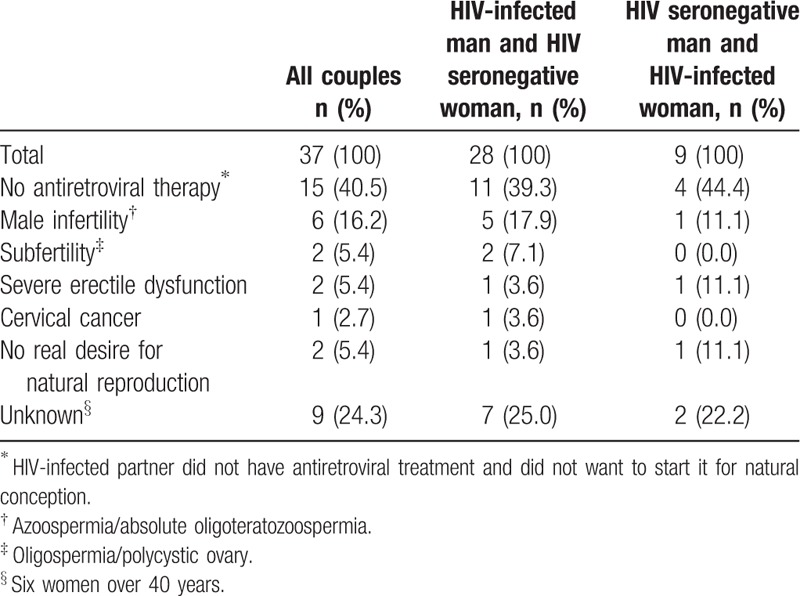
Characteristics of couples who did not opt for natural reproduction.

Table 3 describes the sociodemographic, behavioral, and clinical characteristics of the 161 couples who had unprotected vaginal sex to achieve pregnancy. In 133 (83%) couples, the male was the index case. The main route of HIV infection in women was heterosexual (86%), whereas in men it was injecting drug use (53%), followed by homosexual relations (25%). At the time of seeking reproductive counseling, female index cases had a shorter relationship with their partner, less time since their HIV diagnosis, and less time on ART than male index cases. There were no statistically significant differences by sex in the highest HIV viral load, CD4 nadir, or mean CD4 count at the beginning of the process to achieve reproduction (Table 4).

**Table 3:**
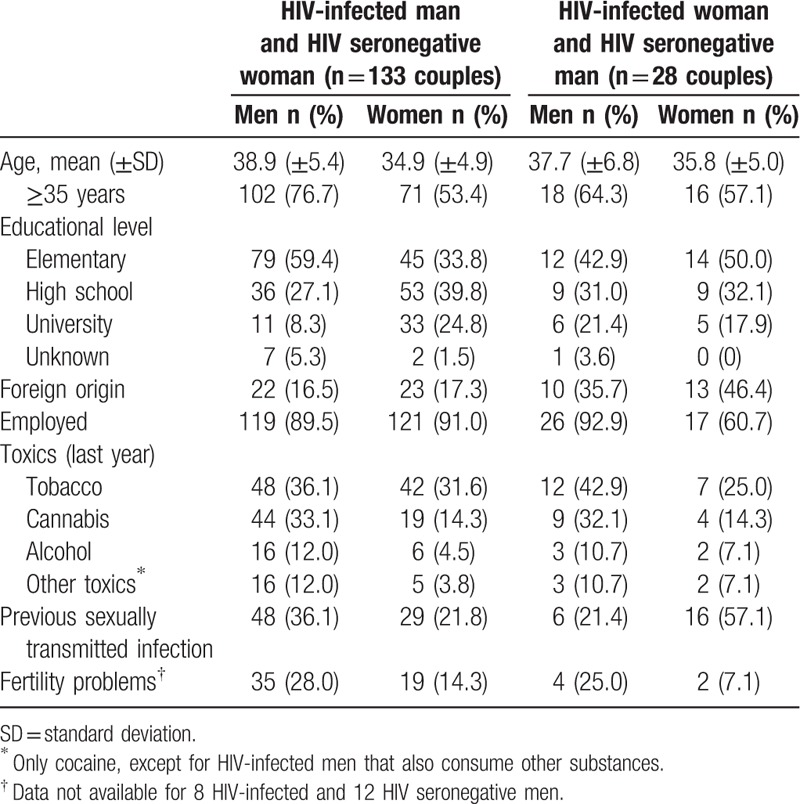
Sociodemographic, behavioral and clinical characteristics of couples who opted for natural reproduction.

**Table 4:**
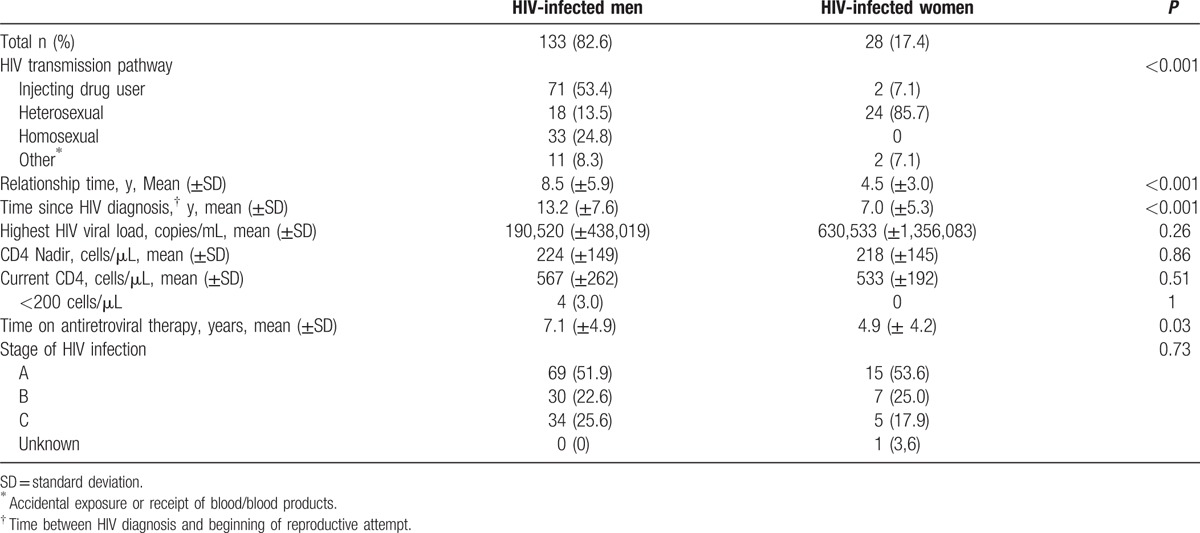
Clinical characteristics of index cases (HIV infected partners).

HIV RNA in semen was undetectable in 100% of the 125 male index cases analyzed. In 8 index cases (6%), however, proviral HIV DNA was detected in semen, and all these men were advised to avoid unprotected sexual relations until they tested negative. In one of these 8 cases *Ureaplasma urealyticum* was identified in an urethral exudate and it was treated with doxycycline 100 mg/12 hours during 10 days. Proviral DNA in semen became negative in a new determination. Another patient, who had begun ART for reproductive purposes, had not achieved sustained viral suppression in plasma (viral load 3153 copies/mL), and tested negative for DNA in semen after achieving undetectable HIV in plasma. The other 6 cases tested negative for proviral DNA in semen in a second determination.

Of the 161 couples who chose natural reproduction, 107 (66%) achieved at least 1 pregnancy, 29 (18%) achieved a second one, and 8 (5%) a third pregnancy. The proportion of couples who achieved at least 1 pregnancy was 65% when the index case was the man versus 71% when it was the woman (*P* = 0.66). Of the 54 couples who had not achieved pregnancy by the end of the study, 28 had been trying for >1 year. The estimated total number of acts of vaginal intercourse for reproductive purposes was 7683, with most of them occurring in couples where the man was the index case. For each 100 acts of vaginal intercourse, there were 1.9 pregnancies: 1.8 pregnancies among HIV-seronegative women and 2.2 among HIV-infected women (*P* = 0.42). The mean time to achieve pregnancy was 6.1 months (standard deviation ± 7.5): 6.4 months in couples with a male index case and 4.3 months when the index case was female (*P* = 0.21). The median of periovulatory acts of vaginal intercourse by couples who achieved pregnancy was 12 (interquartile range [IQR] 5–30), with no significant differences between couples with a male index case (11; IQR 5–30) and those in which the index case was the woman (20; IQR 9–58). A total of 144 pregnancies were achieved: 105 carried to term, 30 spontaneous abortions, 4 voluntary interruptions of pregnancy (2 ectopic pregnancies, 1 Down syndrome, 1 chorioamnionitis post-amniocentesis), 5 cases with an unknown pregnancy outcome. In all, 107 infants were born, and 2 of the births were twins. No cases of sexual or vertical HIV transmission occurred (Table 5).

**Table 5:**
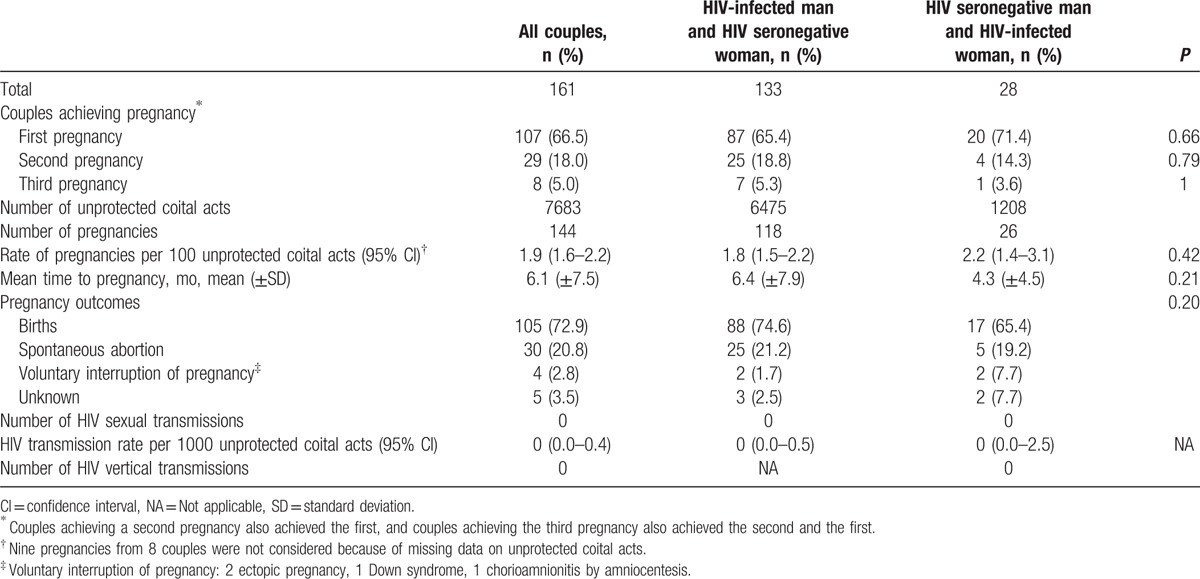
Reproductive results and HIV transmission in couples attempting natural reproduction.

Of the 161 couples who decided to have unprotected sexual relations for the purpose of reproduction, 53 (33%) had some type of clinical-analytic disorder that could affect fertility: 32 in the male (29 male index cases), 14 in the woman (1 woman index case), and 7 in both members of the couple (6 male index case). Table 6 shows the most frequent alterations in semen parameters, and the clinical situations that could alter fertility in the women. Among these 53 couples, there were 32 pregnancies. Seven couples, all with a male index case, had previously undergone assisted reproduction techniques before beginning to attempt natural reproduction: 6 in vitro fertilizations and 1 artificial insemination. Four of the 7 couples achieved pregnancy by the natural method after failing with assisted reproduction.

**Table 6:**
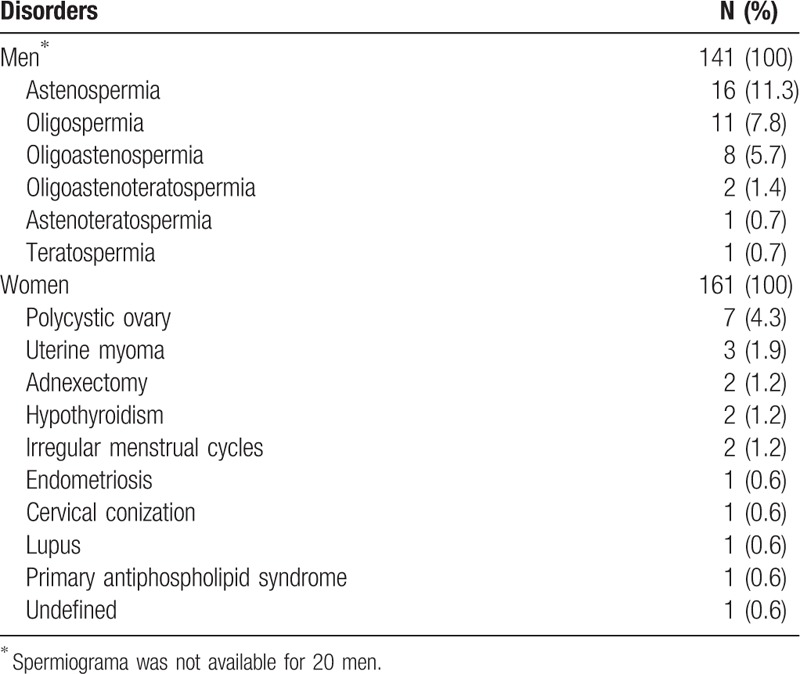
Fertility-related disorders among 161 couples who opted for natural reproduction.

Twelve couples (11 with a male index case) with no identified fertility disorder had made previous attempts to reproduce by artificial insemination (n = 7), in vitro fertilization (n = 3), or both techniques (n = 2). Nine of them achieved pregnancy by the natural method, 4 of whom had not been successful with assisted reproduction.

Four of the 161 couples, 3 with a male index case, 2 of whom had erectile dysfunction, who had programmed unprotected sexual relations for reproductive purposes, combined this practice with autoinsemination, and pregnancy was achieved in 2 cases.

## 4 Discussion

The present study provides the results of a clinical protocol for reproductive counseling aimed at HIV SDCs who wish to have children. A high rate of pregnancies was achieved by natural means with no HIV transmission to either the partner or the offspring after >7000 unprotected sexual relations with ejaculation and 107 births. The fact that 67% of the couples achieved pregnancy shows a high reproductive effectiveness of the program.

A growing number of HIV SDCs are seeking information and advice on reproduction. The limited efficacy of assisted reproduction techniques,^[18,19]^ their high cost, and personal considerations have led a substantial number of couples to consider and attempt natural reproduction without previous professional counseling. Barreiro et al^[8]^ described the outcome of natural pregnancies in SDCs in whom the infected member was receiving suppressive ART. However, the retrospective nature of the study did not allow estimation of the overall risk of HIV transmission, or of reproductive success, as the study analyzed only the couples who achieved pregnancy rather than all who tried to do so. Vernazza et al offered timed intercourse combined with limited preexposure prophylaxis to further reduce the transmission risk to 46 couples, all of them with male index case. Nine of the 46 women decided against the use of preexposure prophylaxis and just performed timed intercourse, mostly because they considered the risk of transmission too low to justify additional drug exposure of the offspring. A total number of 244 unprotected events of vaginal intercourse were documented. The pregnancy rate was of 75% and none of the female partners seroconverted for HIV.^[11]^ A retrospective study in China recognized natural pregnancy as an acceptable option for HIV SDCs in countries with limited resources. The study described 100 natural pregnancies achieved by 91 SDCs, with no cases of horizontal transmission by combining preventive counseling, screening for sexually transmitted infections, effective ART in index cases, autoinsemination in couples wherein the index case was a woman, pre- and post-exposure prophylaxis of seronegative women in couples with a male index case, and programmed periovulatory relations. The authors reported a total of 196 acts of unprotected vaginal intercourses.^[20]^ The couples analyzed in our study are part of a cohort of 930 HIV SDCs in Madrid. The follow-up of this cohort allowed us to document the absence of HIV transmission from infected individuals with suppressive ART to their heterosexual SDCs.^[2,5]^ Detailed data on sexual behavior are systematically collected in this cohort,^[15]^ which has allowed us to document >7000 unprotected events of vaginal intercourse with ejaculation for the purpose of reproduction.

Two-thirds of the couples in our study achieved pregnancy. Actual reproductive effectiveness could be even greater considering that over half of the 54 couples who did not achieve pregnancy had been trying for less than one year. Implementation of the clinical protocol made it possible to identify 33% of couples with potential fertility problems that could affect the probability of achieving a natural pregnancy. Although in some cases assisted reproduction was recommended in accordance with the protocol, it was the couples themselves who finally decided on the reproductive method to use.

Unlike the study of Massad et al,^[21]^ which describes lower pregnancy rates in seropositive than in noninfected women, our study found no differences by sex of the index case in the pregnancy rate per 100 unprotected coital acts. Consistent with those authors, we did not find differences between HIV-infected and seronegative women in the proportion of spontaneous abortions, although no definitive conclusions can be reached given that our series of HIV-infected women was not very large. Couples with a female index case who are considering pregnancy, regardless of the method chosen to achieve it, should be informed that, although their fetuses could potentially be exposed to a greater risk of spontaneous abortion and/or drug-related toxicity,^[22,23]^ the benefits of ART in preventing vertical transmission clearly outweigh these risks.^[7]^

With regard to male index cases, many studies support the thesis that sustained suppression of HIV replication in blood is strongly associated with undetectable HIV RNA in seminal plasma,^[24,25]^ although others describe detection of HIV in seminal plasma in some patients with undetectable viral load in blood.^[9,26–28]^ In our study, we found an absolute correlation between viral load in blood and in semen: in 100% of the men with undetectable plasma viral load on ART, it was also undetectable in semen. It should be noted that our study involved exhaustive screening of and treatment for genitourinary infections before determining the viral load in semen. In our series in 8 index cases, proviral HIV DNA was detected in semen. These men were advised to avoid unprotected sexual relations until they tested negative, all of them belonged to couples who asked for reproductive counseling before 2011. Recently some studies have shown that it is possible to detect integrated HIV DNA in semen cells in long-term ART experienced men.^[29,30]^ It has been pointed intermittent that seminal shedding of HIV (RNA) in spite of suppression of HIV in blood and persistence of HIV DNA in semen should not be considered to place individuals at greater risk for HIV transmission than previously reported.^[26,30]^ So some of the limitations we used in the early years of our program could be dropped in future.

The 2013 French guidelines on reproductive options for people infected with HIV recognize that natural conception is an acceptable reproductive option in SDCs given that ART is a highly effective preventive strategy; the main risk is that poorly informed couples may not respect the conditions that limit the risk of sexual transmission. The guidelines do not recommend pre- and post-exposure prophylaxis in the context of natural conception when there is sustained viral suppression with ART in the index case, unless new studies confirm an additional preventive benefit that is as yet undetermined.^[31]^ The present study might suggest that additional pre- or post-exposure prophylaxis might not be needed, but further studies must be carried out.

This study has several limitations. The number of couples in the study was low to rule out rare events; although we did not detect sexual or perinatal HIV transmission, we cannot totally exclude the risk of HIV transmission under similar conditions. Caution advises us against generalising these results to couples that not totally fulfil the inclusion criteria to the programme. Analysis of self-reported sexual behavior requires some caution. Five couples could not be followed up to the end of the pregnancy; therefore, the transmission to their children cannot be ruled out.

In conclusion, HIV SDCs who wish to have children should receive specialized medical counseling on the appropriate reproductive options in each case. According to our experience, natural pregnancy might be considered, under controlled conditions and in the absence of fertility problems, as a safe and effective method of conception for those HIV SDCs who choose this reproductive option.

## Acknowledgments

The authors thank Dr. B. Menéndez, Dr. D. Carrió, and Dr. E. Nogueira for advice and support.
